# Tumorigenic role of Pak4 in ovarian cancer and its correlation with immune infiltration

**DOI:** 10.1186/s12920-024-01917-4

**Published:** 2024-05-28

**Authors:** Lan Tang, Hong Ye, Li Chen, Weiwei Dong, Xingyan Hu, Lan Yu

**Affiliations:** 1 Department of gynecology, The First College of Medical School, Three Gorges University, Yichang Central People’s Hospital, Yichang, Hubei China; 2 Department of Gynecology and Obstetrics, Guangzhou Women and Children’s Medical Center, Guangzhou Medical University, Guangzhou, China

**Keywords:** Pak4, Tumorigenesis, Ovarian cancer, Survival, Immune infiltration

## Abstract

**Background:**

Ovarian cancer is the most common cause of gynecological cancer death. Pak4 has been proved to be tumorigenic in many types of cancers, but its role in ovarian cancer is still not clarified.

**Methods:**

In this study, we used immunohistochemistry to investigate into Pak4 expression in different histological types of ovarian cancer. TIMER, TISCH2, GEPIA, ualcan, KM plotter, GSCA and GeneMANIA were used to identify the prognostic roles and gene regulation networks of Pak4 in ovarian cancer. Immune infiltration levels were investigated using TIMER database.

**Results:**

Pak4 was highly expressed in ovarian cancers, regardless of different FIGO stages and histological grades. Single cell sequencing database proved that Pak4 was highly expressed in malignant ovarian cancer cells. Pak4 level was significantly correlated with different histological types of ovarian cancer. Pak4 expression was negatively connected with OS and PFS of ovarian cancer patients. Functions of Pak4 and its interacted genes were mainly involved in protein serine/threonine kinase activity, regulation of actin filament-based process and regulation of cytoskeleton organization. Pak4 level was negatively correlated with immune biomarkers of B cell infiltration (*p* = 2.39e-05), CD8 + T cell infiltration (*p* = 1.51e-04), neutrophil (*p* = 1.74e-06) and dendritic cell (*p* = 4.41e-08). Close correlation was found between Pak4 expression and T cell exhaustion (*p* < 0.05).

**Conclusions:**

Our results demonstrated the expression level, gene interaction networks and immune infiltration levels of Pak4 in ovarian cancer. And the results revealed role of Pak4 in tumorigenesis and the possibility to be a potential immunotherapeutic target.

## Introduction

The GLOBOCAN 2020 estimates indicated that new cases of ovarian cancer and number of new deaths reached 313, 959 and 207, 252 respectively worldwide each year [1, 2]. Due to difficulties at early diagnosis, ovarian cancer still remains the most common cause of gynecological cancer death. Although targeted therapeutic drugs have supplemented the treatment methods for ovarian cancer, most ovarian cancer patients suffer from high recurrence challenges [3]. Therefore, it is urgent to search into the mechanism of tumorigenesis and progression and to identify novel sensitive prognostic biomarkers for ovarian cancer patients.

p21-activated serine/threonine kinases (Paks) were effectors for Rho GTPases Rac1 and Cdc42, which were essential in survival, angiogenesis, apoptosis and cytoskeletal reorganization [4]. The Pak4 protein, a member of the Pak family, was first identified necessary for reorganization of the actin cytoskeleton [5]. Furthermore, Pak4 was proved to be associated with proliferation of oesophageal squamous carcinoma cells. It was also found elevated in oesophageal squamous cell carcinoma cells, gastric cancer cells and cervical cancer tissues [6–8]. Researchers also found Pak4 related to cisplatin resistance in cervical cancer and gastric cancer [7, 8]. In triple negative breast cancer cells, reducing Pak4 protein levels also prevented tumor cell growth [9]. In multiple myeloma cells, blocking Pak4 resulted into inhibited myeloma cell growth and survival [10]. In ovarian cancer, Pak4 overexpression could promote cell migration, invasion and proliferation [11]. However, its role in progression and prognosis prediction of ovarian cancer patients still needs intensive research.

Therefore, in this study, we investigated whether Pak4 expression was associated with clinicopathological parameters of ovarian cancer. We also used several bioinformatics tools including Tumor Immune Estimation Resource (TIMER), Tumor Immune Single-cell Hub2 (TISCH2), Gene Expression Profiling Interaction Analysis (GEPIA), Ualcan, Kaplan-Meier (KM) plotter, and Gene Set Cancer Analysis (GSCA) to predict role of Pak4 in prognosis of ovarian cancer patients. To better understand Pak4 regulation network that could affect ovarian cancer prognosis, we preformed GeneMANIA and String analysis. Moreover, we also investigated into the association of Pak4 with tumor immune infiltration and T cell exhaustion using TIMER database. In summary, our study aimed at refreshing the perspectives regarding prognosis prediction and targeted therapy of ovarian cancer.

## Methods

### Immunohistochemistry (IHC)

Tissue samples were commercially obtained from Shanghai Outdo Biotech Company (Shanghai, China). The study was approved by the Ethics Committee of Shanghai Outdo Biotech Company.

The tissue samples were stained with rabbit anti-Pak4 antibody (abcam, ab233215). Staining intensity was categorized as 0 (negative), 1(weak), 2 (moderate) and 3 (strong). The percentage of positive staining was categorized as 0 (< 5%), 1 (5–25%), 2 (26–50%), 3 (51–75%) and 4 (> 75%). The IHC score was calculated as the product of percentage of positive staining intensity and average intensity, with a maximum of 12. Scores ranging from 0 to 2 were considered (-), scores 3–4 were considered (+), scores 5–8 were considered (++) and scores 9–12 were considered (+++). The scores were evaluated by two independent pathologists.

### TIMER

TIMER(https://cistrome.shinyapps.io/timer/)is a database composing 10,897 samples of 32 different cancer types from the Cancer Genome Atlas (TCGA) database. It is usually used for comprehensive analysis of tumor-infiltrating immune cells [12, 13]. In this paper, we analyzed the expression level of Pak4 with TIMER database in different cancer types. The Pak4 expression level was assessed using log2 TPM. TIMER was also used to elucidate role of Pak4 with immune infiltration in ovarian cancer. TIMER2.0 (http://timer.cistrome.org/) was used to analyze the correlation with T cell exhaustion [14].

### TISCH2

TISCH2 (http://tisch.comp-genomics.org/) is a scRNA-seq database. It consists of 190 databases with 6,297,320 single cell data. It provides data at single-cell level to explore the tumor microenvironment among different cancer types [15].

### GEPIA

The online database Gene Expression Profiling Interactive Analysis (GEPIA) database (http://gepia.cancer-pku.cn/) is composed of RNA sequencing expression data of 9,736 tumors and 8,587 normal samples from TCGA and the GTEx database [16]. Pak4 expression data in ovarian cancer vs. normal ovaries, different stages of ovarian cancer were analyzed using GEPIA. P value < 0.05 was recognized statistically significant.

### UALCAN

Ualcan (http://ualcan.path.uab.edu/) is publicly available as a comprehensive web portal which provides online analysis data from TCGA [17]. In this study, we used the Ualcan database to present expression of Pak4 in ovarian cancer based on different cancer stages, tumor grades, patient’s age, patient’s race and TP53 mutation status. Pak4 expression level was normalized as transcript per million reads. *P* < 0.05 conducted through Student’s *t* test was considered statistically significant.

### Kaplan-Meier plotter

The Kaplan Meier plotter (http://kmplot.com) can be used as a tool to assess functions of various genes in different cancer types [18]. In this study, we used the KM plotter to evaluate the progression-free survival (PFS) and overall survival (OS) of Pak4 in ovarian cancers. The prognostic values of high and low Pak4 expression groups in ovarian cancer were represented using hazard ratios (HRs), 95% confidence intervals and logrank P-values. *P* < 0.05 was considered significantly different.

### GSCA

Gene Set Cancer Analysis (GSCA) (http://bioinfo.life.hust.edu.cn/GSCA/#/) is a web-based platform for gene set cancer research [19]. This website offers over ten thousand genomic data among 33 cancer types from TCGA. In this study, we performed survival analysis of PAK4 gene in ovarian cancer using GSCA.

### GeneMANIA

GeneMANIA (http://genemania.org/) is a web portal which analyzes the functions of gene lists [20]. By constructing protein-protein interaction network, GeneMANIA could find neighboring genes associated with targeted gene lists.

### String

String (https://string-db.org/) is a core data resource to collect information for protein-protein interaction using all publicly available sources [21].

### Statistical analysis

Statistical analysis was evaluated using SPSS software (SPSS version 21, SPSS Inc., Chicago, IL, USA). Associations between Pak4 expression and the clinicopathological parameter of ovarian cancer were counted using Chi-square test. GraphPad Prism7.02 (GraphPad Software Inc., San Diego, CA, USA) was used for figure processing. Comparisons between two experimental groups were made using Student’s *t* test. P value < 0.05 was considered statistically significant.

## Results

### Pak4 expression at pan-cancer level using TIMER

**Fig. 1:**
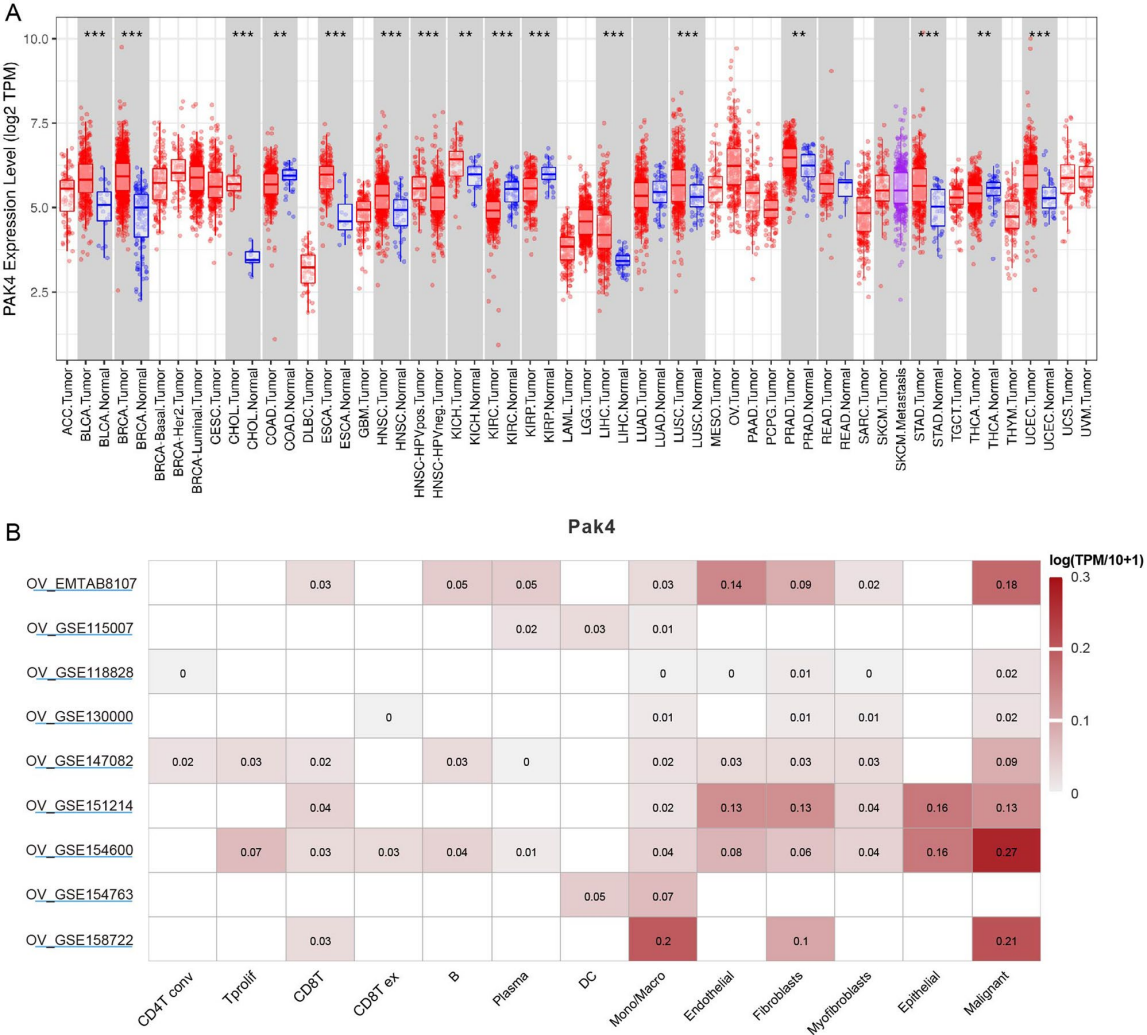
Pak4 expression in various tumors. (**A**) Pak4 expression level was higher in several tumors than in their normal counterparts. **P < 0.01, ***P < 0.001. (**B**) Single cell sequencing proved high Pak4 expression in malignant ovarian cancer cells

In order to investigate into role of Pak4 in different cancers, we used TIMER database to probe into differential expression of Pak4 at pan-cancer level. As shown in Fig. 1A, Pak4 was highly expressed in various tumors, including bladder urothelial carcinoma (BLCA), breast invasive carcinoma (BRCA), cholangiocarcinoma (CHOL), Esophageal carcinoma (ESCA), head and neck cancer (HNSC) and so on. The single cell sequencing database of TISCH2 demonstrated high Pak4 expression in malignant ovarian cancer cells based on several datasets (Fig. 1B). However, Fig. 1A only showed high expression of Pak4 without detailed data in normal ovarian samples, indicating rare investigation of Pak4 in ovarian tissues. Therefore, it is urgent to know Pak4’s role in ovarian cancer tumorigenesis and prognosis.

### Pak4 overexpression correlated with clinicopathological parameters of ovarian cancer

**Fig. 2:**
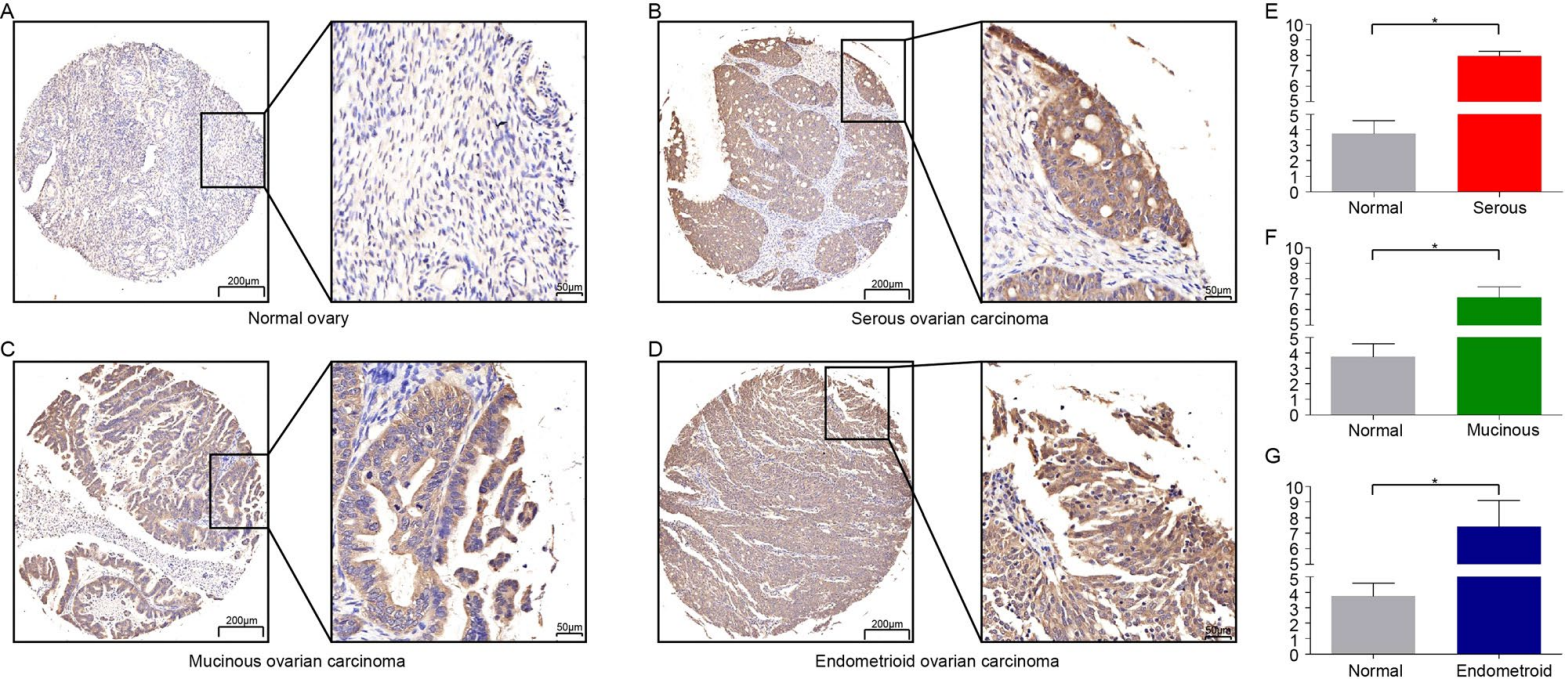
Expression level of Pak4 in ovarian cancer. (**A**) Immunohistochemical staining of Pak4 in normal ovary tissues. (**B**) Immunohistochemical staining of Pak4 in serous ovarian carcinoma. (**C**) Immunohistochemical staining of Pak4 in mucinous ovarian carcinoma. (**D**) Immunohistochemical staining of Pak4 in endometrioid ovarian carcinoma. IHC score of Pak4 in serous (**E**), mucinous (**F**) and endometrioid ovarian carcinoma (**G**)

We investigated Pak4 expression in 163 ovarian cancer samples. The rate of Pak4 staining was represented as -, +, ++, +++, and they were respectively found in 6.7% (11/163), 15.3% (25/163), 37.4% (61/163), 40.5% (66/163) of ovarian cancer patients. Because scores ranging from 5 to 12 were considered high expression, we found Pak4 high expression in 77.9% (127/163) and low expression in 22.1% (36/163) of patients (Table 1). Statistical analyses revealed no significant difference between Pak4 expression and FIGO stages (*P* = 0.339). Histological grades of ovarian cancer did not affect Pak4 expression level (*P* = 0.362). Pak4 demonstrated the highest positive rate in serous cancer (82.4%, 103/125), and the lowest positive rate in mucinous cancer (61.3%, 19/31). Positive rate of Pak4 in endometrioid ovarian cancer was 71.4% (5/7). Pak4 positive rate was statistically significant among different cancer histology types (*P* = 0.037) (Fig. 2A-D; Table 1). The P value between normal ovarian tissue and serous ovarian carcinoma, mucinous ovarian carcinoma and endometrioid ovarian carcinoma in Pak4 expression was 0.0044, 0.01755 and 0.0233, respectively (Fig. 2E-G). In summary, Pak4 expression was statistically different based on histology types instead of FIGO stages or histological grades.

**Table 1:** Correlation of PAK4 with different clinicopathological parameters in ovarian cancer

Characteristics	*n*	Low	High	*P*-value
**FIGO Stage**				0.339
I-II	150	35	115	
III-IV	13	1	12	
**Histological grade**				0.362
1	22	7	15	
2	31	8	23	
3	110	21	89	
**Histology**				0.037*
Serous	125	22	103	
Mucinous	31	12	19	
Endometrioid	7	2	5	

(*P < 0.05)

### Pak4 expression level in ovarian cancer patients

**Fig. 3:**
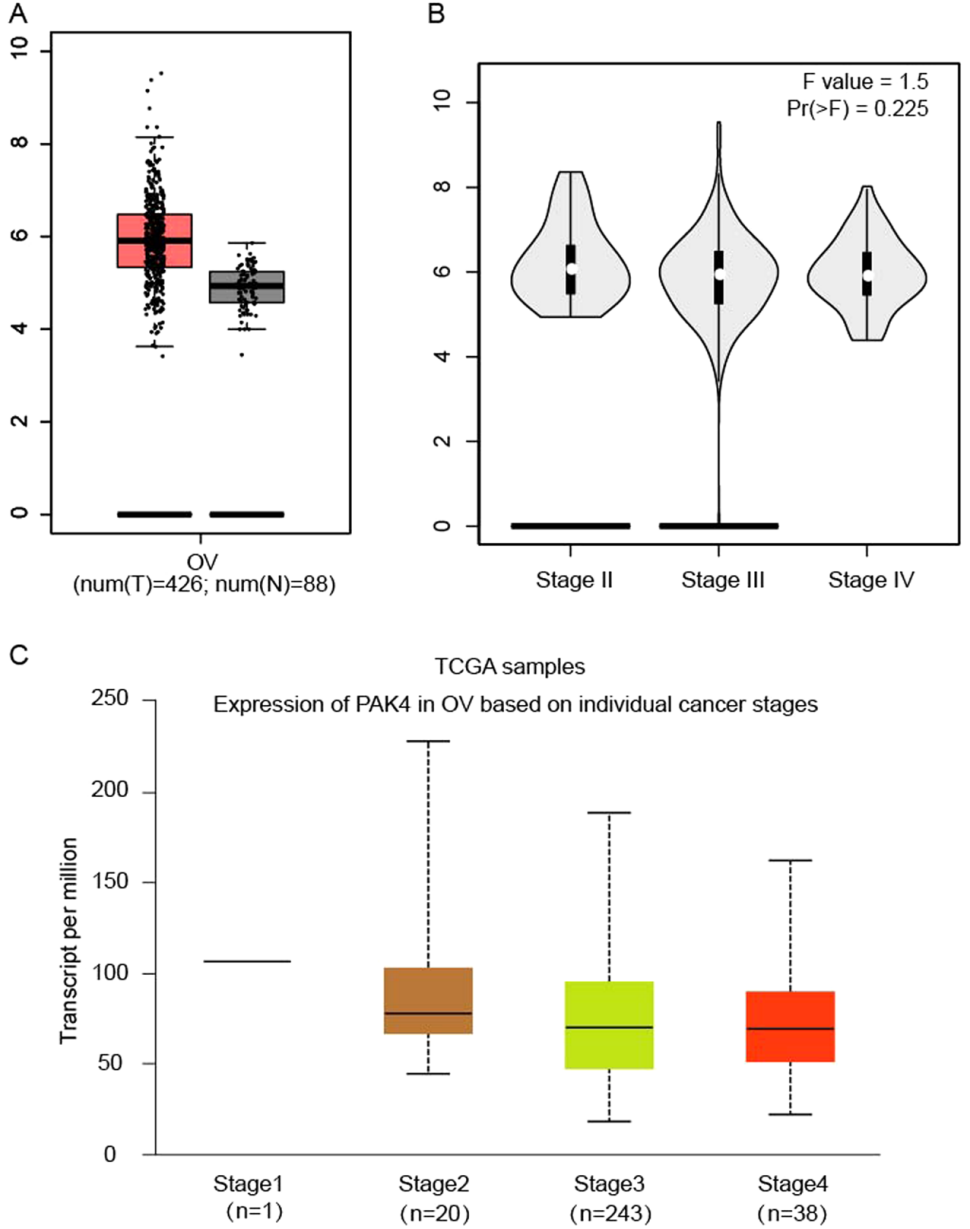
Pak4 expression difference analyzed using GEPIA and ualcan database. (**A**) Pak4 expression level in ovarian cancer and normal ovaries using GEPIA. (**B**) Pak4 expression level evaluated based on different pathological stages using GEPIA. (**C**) Pak4 expression in ovarian cancer based on individual cancer stages using ualcan

**Fig. 4:**
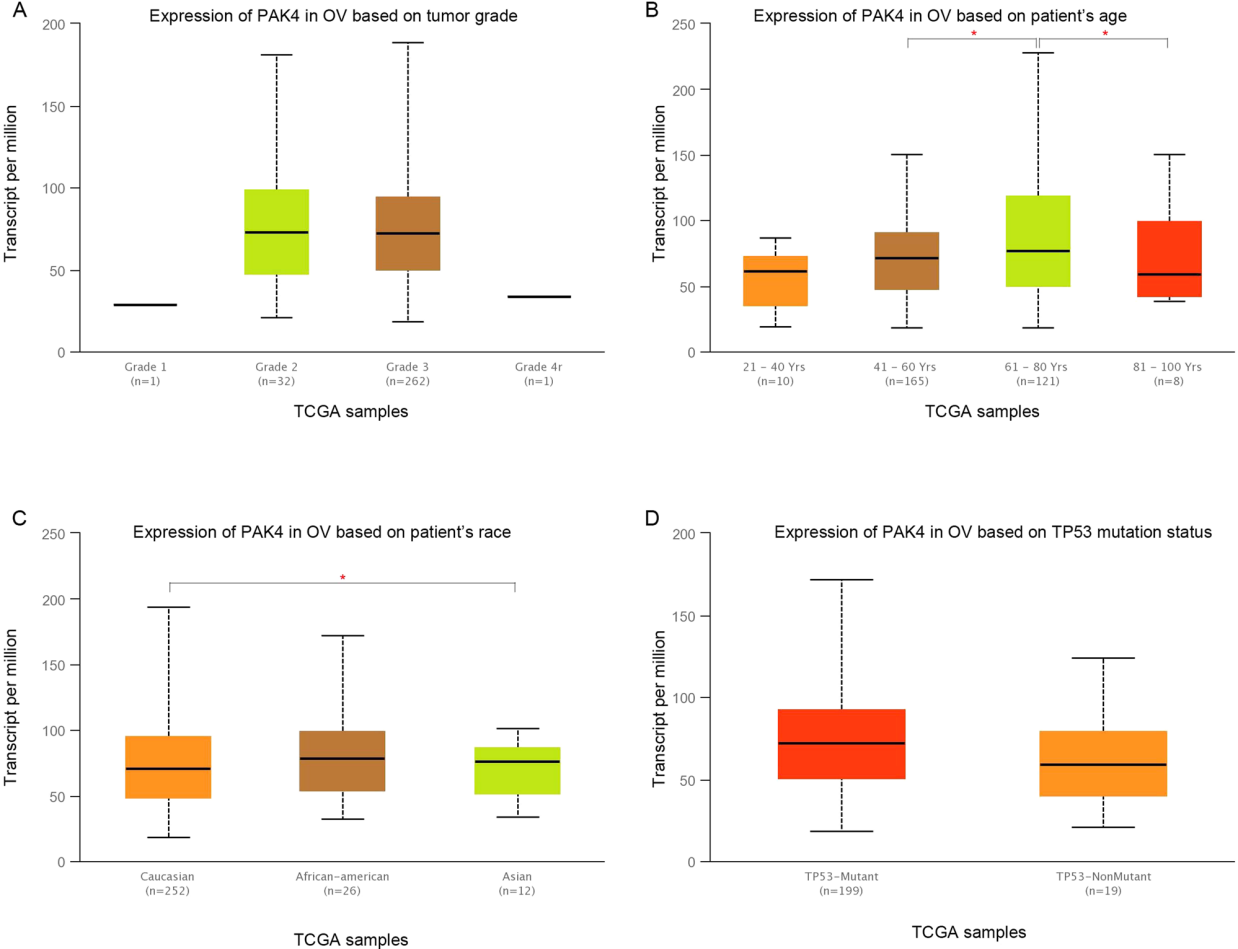
Pak4 expression in ovarian cancer based on different parameters. (**A**) Pak4 expression based on tumor grade. (**B**) Pak4 expression based on patient’s age. (**C**) Pak4 expression based on patient’s race. (**D**) Pak4 expression based on TP53 mutation status

To explore the expression of Pak4 in ovarian cancer, we used GEPIA and Ualcan database. Pak4 expression level in ovarian cancer was higher than in normal ovary tissue using GEPIA (*P* > 0.05; num (T) = 426; num (N) = 88) (Fig. 3A). Although the result did not show significant difference, it could be due to limited number of ovarian cancer patients. Both GEPIA and ualcan database demonstrated no significant difference of Pak4 expression based on individual cancer stages (*P* > 0.05) (Fig. 3B-C). We figured out that Pak4 expression level was not correlated with tumor grade of ovarian cancer (*P* > 0.05) (Fig. 4A). However, Pak4 level was significantly higher among patients aged 61–80 years old (*n* = 121) compared with patients aged 41–60 years old (*n* = 165) (*P* = 4.020300E-03) or patients aged 81–100 years old (*n* = 8) (*P* = 4.564300E-02) (Fig. 4B). We inferred that this might be because of limited number of patients. Pak4 level might be influenced based on patient’s race. For example, Pak4 level was significantly higher in Asian patients (*n* = 12) than in Caucasian patients (*n* = 252)(*P* = 6.964000E-03)(Fig. 4C). However, the number of Asian patients was much less than the number of Caucasian patients in this research. As to TP53 mutation status, it was obtained from TCGA whole exome sequencing data. We found no significant correlation of Pak4 expression between TP53-mutant and TP53 non-mutant patients (*P* = 1.055220E-01) (Fig. 4D).

### The prognostic value of Pak4 in ovarian cancers

**Fig. 5:**
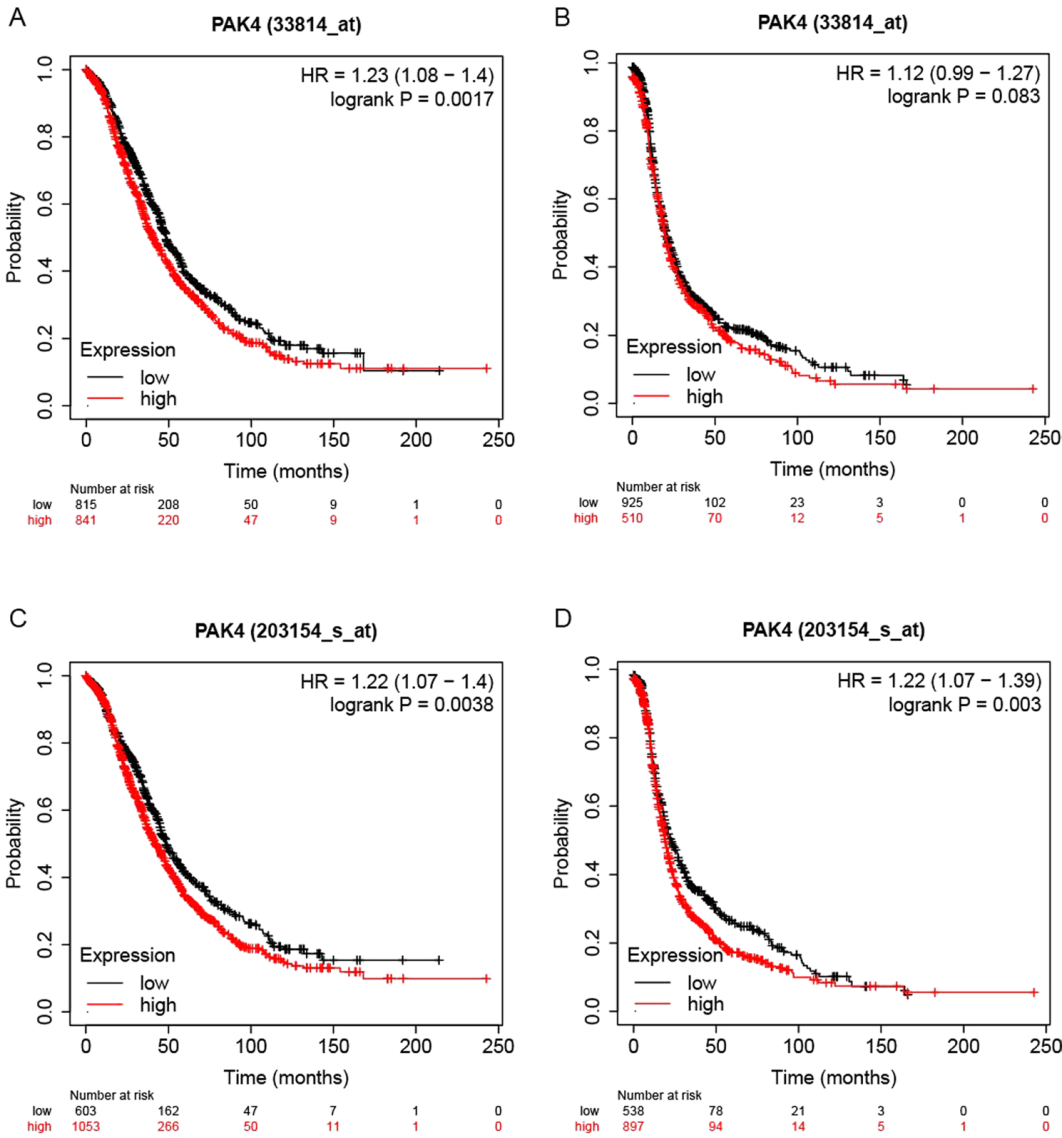
Survival curves of ovarian cancer patients based on Kaplan-Meier plotter. (**A**) OS of patients based on 33814_at probe. (**B**) PFS of patients based on 33814_at probe. (**C**) OS of patients based on 203154_s_at probe. (**D**) PFS of patients based on 203154_s_at probe

The Kaplan-Meier (KM) plotter and GSCA website were implemented for indicating prognostic values of Pak4 expression. Two different microarrays were used to elucidate prognosis of Pak4 in ovarian cancer. We founded that low Pak4 mRNA expression level was correlated with better OS using the 33814_at probe (HR = 1.23 (1.08–1.4), logrank *P* = 0.0017), but no correlation with PFS (HR = 1.12 (0.99–1.27), logrank *P* = 0.083). The 203154_s_at probe showed that low Pak4 expression was correlated with better OS in ovarian cancer (HR = 1.22 (1.07–1.4), logrank *P* = 0.0038) as well as the PFS (HR = 1.22 (1.07–1.39), logrank *P* = 0.003) (Fig. 5).

**Fig. 6:**
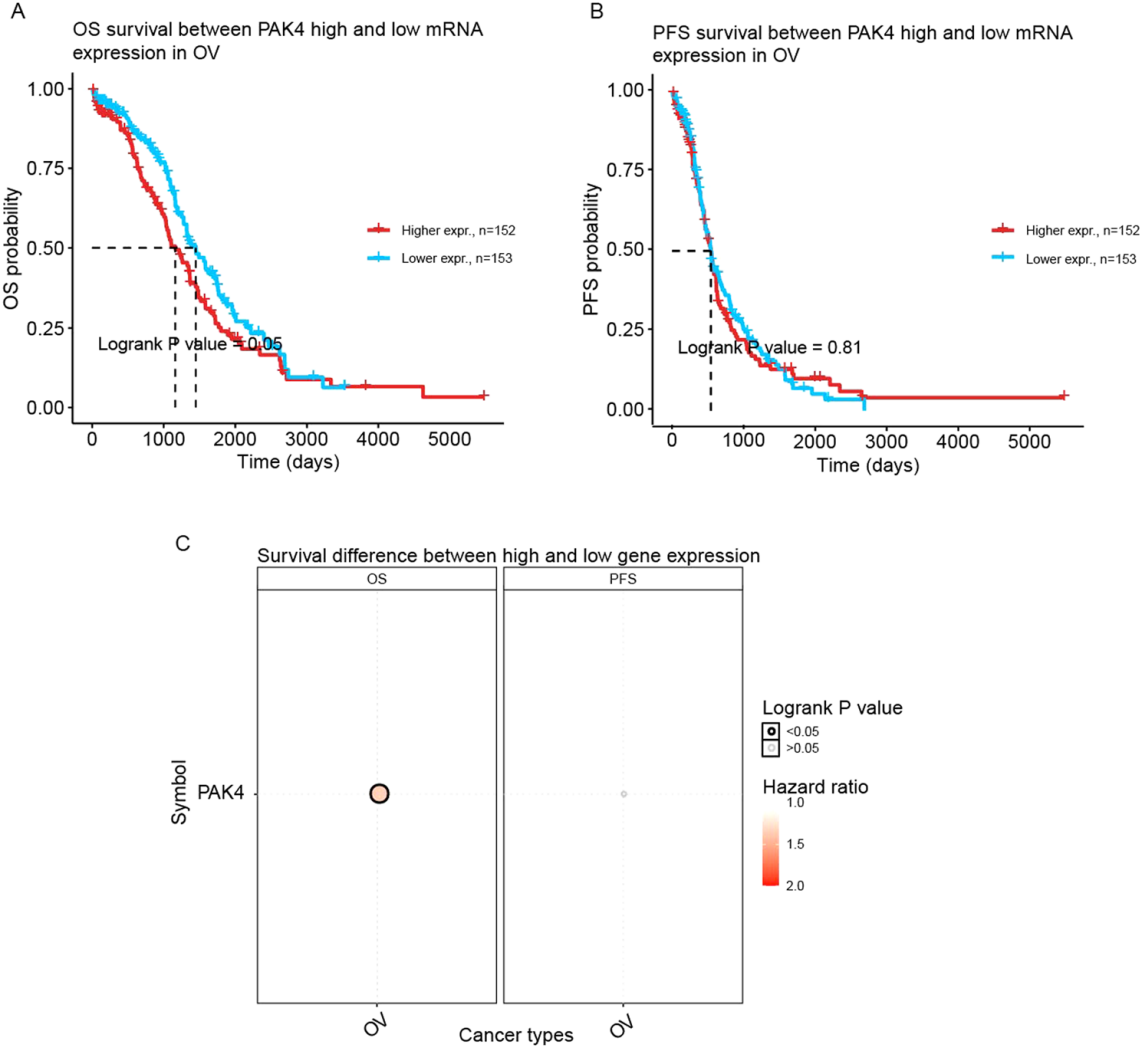
The prognostic value of Pak4 in ovarian cancers based on GSCA. (**A**) OS between Pak4 high and low mRNA expression. (**B**) PFS between Pak4 high and low mRNA expression. (**C**) Survival difference between Pak4 high and low expression

The GSCA website further consolidated the fact that low Pak4 expression level was positively connected with better OS in ovarian cancer (higher expression, *n* = 152; lower expression, *n* = 153) (logrank P value = 0.05) (Fig. 6A and C). However, Pak4 PFS investigated using GSCA did not show any correlation (logrank P value = 0.81) (Fig. 6B). The data suggested that Pak4 might be used as a potential biomarker for predicting prognosis of ovarian cancer patients.

### Construction of a Pak4 based gene-gene interaction network

**Fig. 7:**
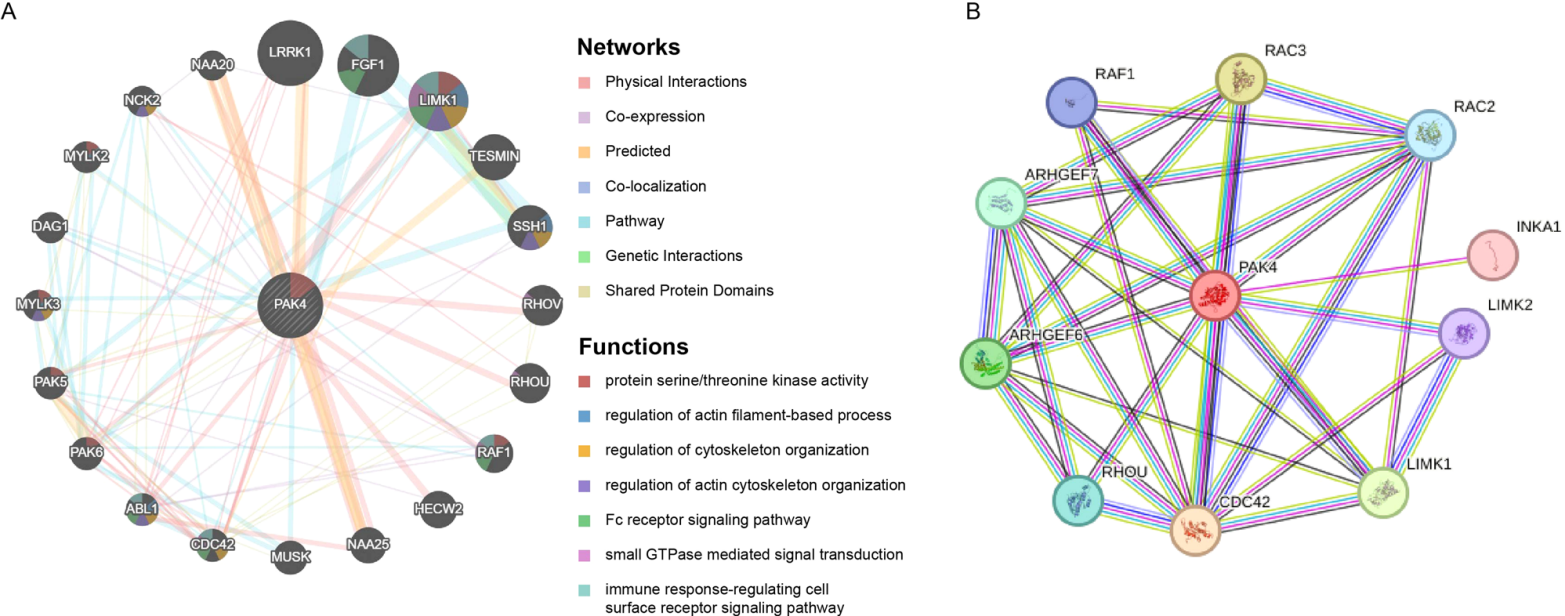
Gene-gene interaction networks of Pak4 gene. (**A**) Each dark circle represents a gene. Size of the dark circle shows strength of interactions. Line colors represent types of gene interaction networks. Colors of circles represent functions of respective genes. (**B**) String database showed Pak4 inter-related gene networks

The interaction networks of Pak4 gene was performed by GeneMANIA and String database. At center of the interaction network, the circle represented Pak4 gene. The Pak4 gene was surrounded by 20 circles representing genes showing close relation with the Pak4 gene based on physical interactions, co-expression, predicted, co-localization, pathway, genetic interactions and shared protein domains (Fig. 7A). The top five genes included the leucine-rich repeat kinase 1 (LRRK1), fibroblast growth factor 1 (FGF1), LIM domain kinase 1 (LIMK1), TESMIN and the (slingshot 1) SSH1 genes, and the five genes displayed greatest correlation with the Pak4 gene. Further analysis showed that these 20 closely correlated proteins were related with functions of protein serine/threonine kinase activity, regulation of actin filament-based process, regulation of cytoskeleton organization, regulation of actin cytoskeleton organization, Fc receptor signaling pathway, small GTPase mediated signal transduction and immune response-regulating cell surface receptor signaling pathway. String database showed several genes partly overlapped with the Genemania database, such as the LIMK1gene, suggesting partial consistency between different database. Therefore, the interaction network needs further investigation (Fig. 7B).

### The correlation between Pak4 expression and immune infiltration

**Fig. 8:**
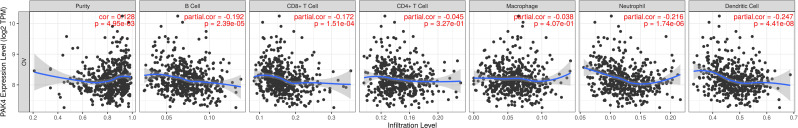
Correlation of Pak4 expression with immune infiltration levels in ovarian cancer. Pak4 expression was positively correlated with tumor purity, negatively correlated with B cell infiltration, CD8 + T cell infiltration, neutrophil and dendritic cell infiltration. No correlation was observed with CD4 + T cell or macrophage infiltration

**Fig. 9:**
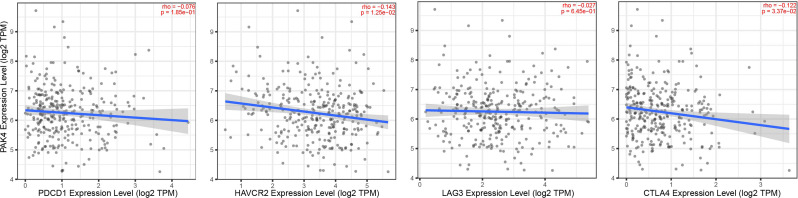
Correlation of Pak4 expression with T cell exhaustion in ovarian cancer

Increasing evidences had shown the importance of immune infiltration in ovarian cancer. Therefore, we investigated whether Pak4 expression showed any correlation with immune cells and immune biomarkers in ovarian cancer based on TCGA data. Pak4 expression showed positive correlation with tumor purity (cor = 0.128, *p* = 4.95e-03), negative correlation with B cell infiltration (partial. Cor=-0.192, *p* = 2.39e-05), CD8 + T cell infiltration (partial. Cor=-0.172, *p* = 1.51e-04), neutrophil (partial. Cor=-0.216, *p* = 1.74e-06) and dendritic cell (partial. Cor=-0.247, *p* = 4.41e-08). No correlation was observed with CD4 + T cell or macrophage infiltration (*p* > 0.05) (Fig. 8). Among all the results, neutrophil and dendritic cell exhibited the highest correlation with Pak4. T cell exhaustion was investigated using TIMER 2.0 database. Pak4 was negatively correlated with HAVCR2 and CTLA4 (*p* = 1.25e-02 and *p* = 3.37e-02), while with no correlation with PDCD1 and LAG3 (*p* > 0.05) (Fig. 9). Therefore, the above results highlighted the key role of Pak4 associated with tumor infiltrating function and T cell exhaustion.

## Discussion

Pak4 has been previously reported to be highly expressed in various cancers [6–9]. However, its role in ovarian cancer has not been extensively studied. In this work, we demonstrated that Pak4 was highly expressed in serous, mucinous and endometrioid ovarian cancer. Pak4 expression level was not correlated to stages or grades of ovarian cancer. More importantly, we found that Pak4 expression level was highly affected by different histological types of ovarian cancer, highlighting importance of ovarian cancer heterogeneity. We further identified Pak4 expression negatively correlated with OS and PFS of ovarian cancer. The above data emphasized the contribution of Pak4 to progression and prognosis outcome of ovarian cancer patients.

We next explored the gene networks correlated to Pak4. We showed that Pak4 expression interacted with LRRK1, FGF1, LIMK1, TESMIN, SSH1 and fifteen other proteins. These proteins closely related with Pak4 were mainly involved with functions of protein serine/threonine kinase activity, regulation of actin filament-based process and regulation of cytoskeleton organization. String showed partially consistent gene networks, suggesting further investigation of Pak4 mechanism in ovarian cancer. Therefore, Pak4 may affect tumorigenesis and prognosis of ovarian cancer patients through interacting with these proteins.

Tumor immune infiltration has gained great attention based on its role in affecting tumor progression and prognosis [22, 23]. It was suggested that Pak4 was overexpressed in biopsies of patients with low T cell infiltration. Low T cell infiltration was reported to be related to resistance to programmed cell death protein1 (PD-1) blockade. Therefore, Pak4 overexpression may induce PD-1 blockade treatment failure. Genetic deletion of PAK4 was reported to increase T cell infiltration and reverse the resistance to PD-1 blockade. Inhibiting PAK4 might contribute to better response towards anti-tumor treatment [24]. In our study, we also investigated the relation of Pak4 with tumor infiltration level and T cell exhaustion using TIMER database. We found that Pak4 level was negatively correlated with B cell infiltration, CD8 + T cell infiltration, neutrophil and dendritic cell infiltration. These results suggested oncogenic roles of Pak4 in ovarian cancer. Pak4 was also related with T cell exhaustion according to our results, but the mechanism needs to be clarified with further research (Fig. 9). Thus, Pak4 may play an important role in T cell exhaustion and immune escape of ovarian cancer microenvironment.

In summary, our study proved that Pak4 was highly expressed in ovarian cancer. Pak4 level significantly correlated with histological parameters and prognosis of ovarian cancer patients. Pak4 was negatively associated with OS curves of ovarian patients, suggesting its oncogenic role. Pak4 and its interacting genes were primarily involved in protein serine/threonine kinase activity, regulation of actin filament-based process and regulation of cytoskeleton organization. Pak4 level correlated closely with immune infiltration and T cell exhaustion in ovarian cancer microenvironment. All of the above results suggested that Pak4 played vital roles in ovarian cancer tumorigenesis and progression. Therefore, Pak4 may be used as a promising prognostic biomarker for ovarian cancer.

## Data Availability

All the data and results generated during the study are available from the corresponding author on reasonable request.

## Abbreviations

GEPIA: Gene Expression Profiling Interaction Analysis
TIMER: Tumor Immune Estimation Resource
KM plotter: Kaplan-Meier plotter
PFS: progression-free survival
OS: overall survival
HRs: Hazard ratios
